# Progesterone after previous preterm birth for prevention of neonatal respiratory distress syndrome (PROGRESS): a randomised controlled trial

**DOI:** 10.1186/1471-2393-9-6

**Published:** 2009-02-24

**Authors:** Jodie M Dodd, Caroline A Crowther, Andrew J McPhee, Vicki Flenady, Jeffrey S Robinson

**Affiliations:** 1 Discipline of Obstetrics and Gynaecology, The University of Adelaide, Adelaide, SA, Australia; 2 Department of Neonatology, The Women's and Children's Hospital, Adelaide, SA, Australia; 3 Centre for Clinical Studies, Mater Health Services, Brisbane, SA, Australia

## Abstract

**Background:**

Neonatal respiratory distress syndrome, as a consequence of preterm birth, is a major cause of early mortality and morbidity during infancy and childhood. Survivors of preterm birth continue to remain at considerable risk of both chronic lung disease and long-term neurological handicap. Progesterone is involved in the maintenance of uterine quiescence through modulation of the calcium-calmodulin-myosin-light-chain-kinase system in smooth muscle cells. The withdrawal of progesterone, either actual or functional is thought to be an antecedent to the onset of labour. While there have been recent reports of progesterone supplementation for women at risk of preterm birth which show promise in this intervention, there is currently insufficient data on clinically important outcomes for both women and infants to enable informed clinical decision-making.

The aims of this randomised, double blind, placebo controlled trial are to assess whether the use of vaginal progesterone pessaries in women with a history of previous spontaneous preterm birth will reduce the risk and severity of respiratory distress syndrome, so improving their infant's health, without increasing maternal risks.

**Methods:**

**Design: **Multicentred randomised, double blind, placebo-controlled trial.

Inclusion Criteria: pregnant women with a live fetus, and a history of prior preterm birth at less than 37 weeks gestation and greater than 20 weeks gestation in the immediately preceding pregnancy, where onset of labour occurred spontaneously, or in association with cervical incompetence, or following preterm prelabour ruptured membranes.

Trial Entry & Randomisation: After obtaining written informed consent, eligible women will be randomised between 18 and 23^+6 ^weeks gestation using a central telephone randomisation service. The randomisation schedule prepared by non clinical research staff will use balanced variable blocks, with stratification according to plurality of the pregnancy and centre where planned to give birth. Eligible women will be randomised to either vaginal progesterone or vaginal placebo.

Study Medication & Treatment Schedules: Treatment packs will appear identical. Woman, caregivers and research staff will be blinded to treatment allocation.

Primary Study Outcome: Neonatal Respiratory Distress Syndrome (defined by incidence and severity).

Sample Size: of 984 women to show a 40% reduction in respiratory distress syndrome from 15% to 9% (p = 0.05, 80% power).

**Discussion:**

This is a protocol for a randomised trial.

**Clinical Trial Registration:**

Current Controlled Trials ISRCTN20269066

## Background

### Respiratory distress syndrome: the burden of disease

Neonatal respiratory distress syndrome, secondary to immature pulmonary development, is a major cause of infant mortality and morbidity among infants born preterm [1]. Infants born preterm often require mechanical ventilation [2], with up to 20% of survivors remaining oxygen dependent at 28 days of age, and 25% being diagnosed with chronic lung disease [2]. Additionally, infant who are born preterm have a recognised increased risk of hospitalisation within their first year of life[3], and considerable longer-term risk of neurological handicap, including cerebral palsy[4].

Birth before 37 weeks gestation occurred in 7% of pregnancies within Australia during 2002[5], with 2.6% of all births occurring prior to 34 weeks gestation [6]. However, this relatively small proportion of total births within Australia, accounts for almost 70% of the total perinatal mortality[6]. It has been estimated that 50% of preterm births occur in the setting of spontaneous preterm labour, or following preterm ruptured membranes [7,8].

The financial costs associated with preterm birth and subsequent admission of the infant to the intensive care nursery are not inconsiderable, with estimates from the United States from 1990 suggesting a weekly cost of approximately $US10,000 per preterm baby, or in excess of $5 billion annually[9]. Not surprisingly, the cost of care increases for infants of lower birthweight, estimated to be $140,000 where the birthweight is less than 1000 grams[9]. Those infants who suffer severe disability have long-term care costs estimated to be more than $100,000 and the cost of lifetime custodial care has been estimated to reach $450,000[9]. These monetary estimates do not take account of both the emotional and personal costs to the family and individuals themselves, which are considerable.

### The likelihood of recurrence of preterm birth

Population cohort data suggests that women who have had a previous preterm birth are more likely to give birth preterm in a subsequent pregnancy [10-12], with up to a third of women giving birth prior to 37 weeks gestation[12]. For 8.2% of women with a previous preterm birth, subsequent infants will be born at a similar gestational age[12]. Other authors have estimated the rate of recurrent preterm birth to be 22.5%[13], which represents a two and a half fold increase in the risk of preterm birth, when compared with women who have not had a previous spontaneous preterm birth[14].

### Respiratory distress syndrome: How can we reduce the burden of disease?

Reducing an infant's risk of respiratory distress syndrome requires prevention of preterm birth or interventions to improve fetal and neonatal lung maturation. To date, interventions to prevent preterm birth remain elusive. It has been estimated that in developed nations, the rate of preterm birth has remained relatively constant between 6 and 10% for the past four decades, despite a multitude of intervention strategies aimed at various 'at-risk' populations.

Studies involving the use of progesterone as an agent to prevent preterm birth date to the 1960's [15], although with the recent publication of randomised trials, there has been renewed interest in its use in pregnancy.

### The role of progesterone in the initiation of labour

The onset of labour in women is a complex interaction of different hormonal pathways [16-19], with progesterone being considered essential to the maintenance of a normal healthy pregnancy [20-22]. Progesterone has an important role in maintaining uterine quiescence[23,24], acting to reduce calcium flux of smooth muscle through suppression of the calcium-calmodulin-myosin light chain kinase system[19,21].

There is debate about the previously described progesterone withdrawal[25] as an antecendent to the onset of labour[26]. Seven progesterone receptor subtypes have been classified in women, with alterations in the ratio of receptor expression resulting effectively in withdrawal of progesterone[26,27], and a resultant increase in the sensitivity of the myometrium to contractile stimuli[20,22,28,29].

Documented in many animals is a reduction in serum progesterone concentrations prior to the onset of labour [16,19,20,30]. However, this has not been shown to occur before term or preterm labour in women, with no detectable changes in the concentrations of steroid hormones[19,20,22,31,32]. However, the pharmacological withdrawal of progesterone with inhibitors such as mifepristone (RU486), has been used clinically to interrupt pregnancy and facilitate termination [33,34], while progesterone has been used to maintain pregnancy in women undergoing assisted reproductive techniques, until placental production suffices[35].

Progesterone is also known to have anti-inflammatory properties, raising a potential link between inflammatory processes, alterations in progesterone receptor expression and onset of preterm labour [36].

### Safety of progesterone

The use of natural progesterone in pregnancy has been considerable, without documented effect on the risk of congenital anomalies or other aspects of fetal development[37,38]. Animal studies in sheep have suggested that prolonged exposure to high concentrations of progesterone may affect fetal behavioural states[39], with suppression of activity and arousal[40,41]. Further information about the effect of prolonged exposure of the fetus to progesterone therapy during pregnancy is required.

Maternal side effects related to progesterone therapy are minor and of a temporary nature, including headache, nausea, breast tenderness, and coughing.

### The clinical evidence for the use of progesterone in the prevention of preterm birth: The Cochrane Review

The use of progesterone in pregnancy for the prevention of preterm birth has been evaluated in a Cochrane systematic review[42], in which seven randomised trials were identified comparing intramuscular 17 alphahydroxyprogesterone caproate with placebo[15,43-48]. Two trials were excluded as they adopted a quasi-randomised method of treatment allocation[15,47]. In a meta-analysis of these five trials, women who received progesterone had a statistically significant lower risk of preterm birth and in keeping with this, fewer infants with birth weight less than 2.5 kg when compared with those women administered placebo[42]. There was insufficient evidence to assess the effects of progesterone therapy on perinatal death or respiratory distress syndrome, with a single trial only[45] reporting information regarding neonatal morbidity. However, intra-muscular 17 alpha-hydroxyprogesterone caproate is not available for general prescription in Australia, North America, or the United Kingdom.

A single randomised trial was identified comparing vaginal progesterone pessaries with placebo[49], in which 157 Brazilian women with a singleton pregnancy considered to be at "high risk" of preterm birth (history of previous preterm birth, the presence of a cervical suture, or uterine malformation), were recruited. Women were randomised to receive either 100 mg progesterone or placebo suppositories administered nightly between 24 and 34 weeks gestation[49]. While there were fewer women in the progesterone group who gave birth before 37 weeks gestation (10/72 (13.9%) progesterone versus 20/70 (28.6%) placebo; relative risk 0.49; 95% Confidence Intervals 0.25–0.96; p = 0.03), there were no other maternal or infant health outcomes reported[49].

### To summarise

There are limitations of the current evidence on the use of vaginal progesterone for women at increased risk of preterm birth, including the lack of reporting of maternal and infant outcomes; the lack of information about potential harmful fetal and infant effects related to in-utero exposure to progesterone related to the use of vaginal progesterone.

### Aims of the trial

The aims of this randomised, double blind, placebo controlled trial are to assess whether the use of vaginal progesterone pessaries in women with a history of previous spontaneous preterm birth will reduce the risk and severity of respiratory distress syndrome, so improving their infant's health, without increasing maternal risks.

### Trial hypotheses

**The primary hypothesis **of this randomised trial is that the administration of progesterone to women with a history of previous spontaneous preterm birth will

**• reduce the risk of neonatal respiratory distress syndrome**.

**The secondary hypotheses **of this randomised trial are that the administration of progesterone to women with a history of previous spontaneous preterm birth will

**• reduce the risk of morbidity from neonatal lung disease**;

• reduce the risk of morbidity from other adverse outcomes for the infant; and

**• reduce the risk of maternal morbidity without adverse effects of therapy**.

## Methods/Design

### Study Design

Multicentred randomised, double blind, placebo-controlled trial.

### Inclusion Criteria

Pregnant women with a live fetus confirmed at the time of trial entry between 18 and 23^6 ^weeks gestation, who have a history of prior preterm birth at less than 37 weeks gestation and greater than 20 weeks gestation (either vaginal birth or caesarean birth) in the immediately preceding pregnancy, where the onset of labour occurred spontaneously, or in association with cervical incompetence, or following preterm prelabour ruptured membranes. Women who receive progesterone therapy early for early pregnancy support (prior to 16 weeks gestation) in the current pregnancy will be eligible for trial inclusion.

### Exclusion Criteria

Women with the following will not be eligible for participation:

• Women whose immediately preceding preterm birth at less than 37 weeks gestation was associated with:

○ Placental abruption or placenta praevia

○ Multiple pregnancy

○ Iatrogenic decision for early birth (for example related to fetal distress, preeclampsia, eclampsia)

• Women whose current pregnancy is associated with:

○ Active vaginal bleeding requiring hospital admission after 17^+6 ^weeks of gestation

○ Current preterm prelabour ruptured membranes diagnosed prior to trial entry

○ Active labour (defined as the presence of uterine activity and cervical dilatation greater than 3 cm)

○ Known lethal fetal anomaly

○ Fetal demise

○ Progesterone treatment during the current pregnancy after 16 weeks gestation

○ Any contraindication to continuation of the pregnancy (eg. chorioamnionitis requiring delivery)

○ Any contraindication to progesterone therapy (known active liver disease, thrombophlebitis, active thromboembolism (or hormone related), breast or genital malignancy).

The use of progesterone in the following conditions is not contraindicated but the manufacturers recommend use with monitoring: serious depression and medical conditions that may be aggravated by fluid retention (asthma, epilepsy, migraine, known cardiac dysfunction, known renal dysfunction).

### Trial Entry

Eligible women will be identified in the antenatal clinic, given the trial information sheet and counselled by the researcher, before obtaining informed written consent. Randomisation will occur between 18 and 23^+6 ^weeks gestation by telephoning the cental randomisation service. The randomisation schedule will use balanced variable blocks, and will be prepared by an investigator not involved with recruitment or clinical care. There will be stratification of women according to plurality of the pregnancy (singleton versus twin pregnancy versus triplet pregnancy) and centre. Eligible women will be randomised to either vaginal progesterone therapy or vaginal placebo.

### Approval to conduct this study has been obtained from the following research and ethics committees

Women's and Children's Hospital (Adelaide, South Australia); Modbury Public Hospital (Adelaide, South Australia); Flinders Medical Centre (Adelaide, South Australia); Lyell McEwin Health Service (Adelaide, South Australia); The Queen Elizabeth Hospital (Adelaide, South Australia); Mercy Health and Aged Care (Melbourne, Victoria); Royal Women's Hospital (Melbourne, Victoria); Monash Medical Centre (Melbourne, Victoria); Sydney West Area Health Service (New South Wales); Sydney South Area Health Service (New South Wales); Northern Sydney Central Coast (New South Wales); Sydney Adventist Hospital (New South Wales); ACT Health (Australian Capital Territory); Mater Health Services (Brisbane, Queensland); Toowoomba Health Service (Queensland); Ipswich Hospital (Queensland); Redcliffe-Caboolture Health Service (Queensland); Townsville Health Service (Queensland); Launceston General Hospital (Tasmania); Christchurch Women's Hospital (New Zealand); Auckland Hospital (New Zealand); Mt Sinai Hospital (Toronto, Canada).

### Study Medication & Treatment Schedules

After randomisation, the woman will be allocated a study number that corresponds with the same number on her treatment pack. The woman, her caregivers and research staff assessing the trial outcomes will be blinded to treatment allocation. Treatment packs will appear identical for the treatment groups. Women will be asked to self-administer equivalent of 100 mg vaginal progesterone each evening from 20 weeks gestation, or randomisation (if this occurs after 20 weeks gestation) until birth or 34 weeks gestation (whichever occurs first). Women who develop prelabour ruptured membranes after trial entry will remain in their treatment groups for the purposes of analysis, but will be advised to discontinue using vaginal pessaries to reduce the risk of introducing vaginal or ascending infection.

### Follow-up of women in both treatment groups

Women will be reviewed in the antenatal clinic according to the recommendation of the practitioner responsible for their care. Women will receive further trial medication treatment packs on a monthly basis. At 34 weeks gestation, women will be asked about the occurrence of any side effects experienced and compliance with the treatment protocol. After birth, information will be obtained relating to birth and infant outcomes from the woman's and infant's case notes by the research assistant and the delivery form completed. Similarly, the postnatal and neonatal forms will be completed for each live born infant after discharge from hospital.

### Primary Study Endpoints

The primary study outcome is:

**• Neonatal Respiratory Distress Syndrome **(defined by the incidence (increasing respiratory distress or oxygen requirement or the need for respiratory support from the first six hours of life, in a term or preterm infant) and severity of neonatal respiratory disease (mild = mean airway pressure (MAP) < 7 cm H_2_O, and/or fractional inspired oxygen (FiO_2_) < 0.4; moderate = MAP 7–9.9 cm H_2_O, and/or FiO_2 _0.40–0.79; severe = MAP ≥ 10 cm H_2_O, and/or FiO_2 _≥ 0.80 with need for ventilation)[2].

### Secondary Study Endpoints

The secondary study endpoints are:

**1) Other respiratory outcomes **defined as: main respiratory diagnosis (that is, the main indication for respiratory support for the baby), need for and duration of oxygen therapy (including highest FiO_2 _(%) within 12 hours birth), and need for and duration of mechanical ventilation (including maximum peak pressure (cm H_2_O) within 12 hours birth), air leak syndrome, need for surfactant therapy, nitric oxide for respiratory support, and chronic lung disease (defined as the need for any respiratory support, supplemental oxygen or intermittent positive pressure ventilation or continuous positive airways pressure for a chronic pulmonary disorder on the day the baby reached 36 weeks' postmenstrual age, for infants born before 32 weeks gestation, or continued oxygen requirement at 28 days of age for infants born after 36 weeks gestation).

**2) Adverse outcomes for the infant **defined as one or more of the following: preterm birth (defined as birth at less than 37 weeks gestation) and mortality (defined as either a stillbirth (intrauterine fetal death after trial entry and prior to birth), or infant death (death of a live born infant prior to hospital discharge, and excluding lethal congenital anomalies)); Apgar score < 4 at 5 minutes of age; birth weight less than the 3rd centile for gestational age at birth and infant sex; intraventricular haemorrhage on early cranial ultrasound; periventricular leucomalacia on later cranial ultrasound; inotropic support for the treatment of patent ductus arteriosus; proven necrotising enterocolitis; proven systemic infection within 48 hours of birth and treated with antibiotics; retinopathy of prematurity.

**3) Adverse outcomes for the woman **including length of antenatal hospital stay; use of tocolytic therapy; antenatal corticosteroid therapy; side effects of progesterone supplementation (including headache, nausea, breast tenderness, coughing); antepartum haemorrhage; pre-eclampsia; preterm prelabour ruptured membranes; prelabour ruptured membranes at or near term (defined as prelabour ruptured membranes after 36 weeks gestation); chorioamnionitis requiring antibiotic use during labour; postpartum haemorrhage; antibiotic use after birth; length of postnatal hospital stay; maternal death; not breast feeding at four months postpartum.

### Sample Size

The clinical endpoint of respiratory distress syndrome has been chosen as the primary endpoint. For women eligible for this trial, the best estimate of the incidence of respiratory distress syndrome is 15%, using information from a randomised trial with similar eligibility profile[45]. A sample size of 984 women will be able to show a 40% reduction in respiratory distress syndrome from 15% to 9% (5% level of significance, two-tailed alpha, 80% power).

### Analysis and Reporting of Results

The initial analysis will examine baseline characteristics of all randomised women, as an indication of comparable treatment groups. Outcome comparisons for women and infants will be analysed for the primary and secondary outcomes on an "intention to treat" basis, according to treatment allocation at randomisation to either progesterone or placebo. The relative risks and 95% confidence intervals will be reported for the major outcomes, and the number needed to treat to prevent one adverse outcome will be calculated. Regression techniques will be used to examine the influence of prognostic factors on the major outcomes.

## Discussion

This is a protocol for a randomised trial assessing the role of vaginal progesterone in women with a previous spontaneous preterm birth. The findings of this trial will contribute to the currently available literature regarding the use of vaginal progesterone.

## Competing interests

The authors declare that they have no competing interests.

## Authors' contributions

JMD, CAC, AJM, VF, JSR all contributed to the development of the trial protocol. JMD drafted the manuscript and all authors reviewed critically for content and gave approval to the final to be published version of the manuscript.

## Pre-publication history

The pre-publication history for this paper can be accessed here:


